# Rethinking the 2-minute rule in adult basic life support cardiopulmonary resuscitation

**DOI:** 10.1016/j.resplu.2025.101070

**Published:** 2025-08-23

**Authors:** Emma Menant, Guillaume Debaty, Janet Bray, Thomas Rea, Xavier Jouven

**Affiliations:** a Université Paris Cité, Inserm, PARCC, F-75015 Paris, France; b Paris Cardiac Arrest Center, European Georges Pompidou Hospital, APHP, Paris, France; c Emergency Department and Mobile Intensive Care Unit, University Hospital of Grenoble Alpes, Grenoble, France; d Univ. Grenoble Alpes, CNRS, UMR 5525, VetAgro Sup, Grenoble INP, TIMC, 38000 Grenoble, France; e School of Public Health and Preventive Medicine, Monash University, Melbourne, Australia; f Prehospital, Resuscitation and Emergency Care Research Unit (PRECRU), School of Nursing, Midwifery and Paramedicine, Curtin University, Bentley, Australia; g Department of Medicine, University of Washington, Seattle, WA, United States; h King County Emergency Medical Services Seattle-King County Department of Public Health Seattle WA, United States

**Keywords:** Out-of-hospital cardiac arrest, Cardiopulmonary resuscitation, Automated external defibrillator, Guidelines, Recommendations, Rhythm analysis, Chest compression fraction, Ventricular fibrillation duration

## Abstract

The standard 2-minute cycle of cardiopulmonary resuscitation (CPR) followed by rhythm analysis has long guided out-of-hospital cardiac arrest basic life support. However, recent studies suggest this method may not optimize return of spontaneous circulation. Research shows most ventricular fibrillation recurrences occur within a minute post-shock, supporting earlier defibrillation and rhythm analysis during CPR to improve survival. Conversely, when no shock is needed, longer CPR durations may be more beneficial. This commentary article reviews the evolution of CPR guidelines and critically assesses new evidence, advocating for a re-evaluation of the 2-minute interval in favour of more flexible, patient-specific resuscitation strategies.

## Introduction

**Fig. 1:**
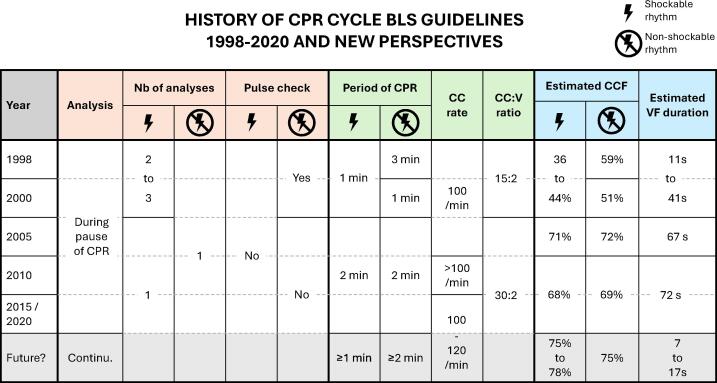
Changes of the analysis/CPR cycle from 1998 to 2015, considering the changes in adult basic life support guidelines (AHA/ERC), with a proposition for future. Chest compression fraction and ventricular fibrillation duration are estimated in Additional file. AHA: American Heart Association; BLS: Basic Life Support; CC: Chest Compression; CCF: Chest Compression Fraction; CPR: Cardiopulmonary Resuscitation; ERC: European Resuscitation Council; V: Ventilation; VF: Ventricular Fibrillation.

In cardiopulmonary resuscitation (CPR), the standard practice of performing chest compressions (CC) and ventilation for two minutes between rhythm analysis is firmly established in international basic life support (BLS) guidelines.^1^, ^2^ The two-minute algorithm was formally adopted in the 2005 guidelines to optimise CC delivery and training. However, the studies supporting this duration do not reflect current practice. Since 2005, several guideline updates have resulted in positive changes, such as increased chest compression fraction (CCF).^3^ However, these improvements may have also unintentionally prolonged the duration of untreated ventricular fibrillation (VF) (Fig. 1). Recent advances in defibrillation technology and rhythm detection challenge the continued need for a fixed 2-minute cycle. Given these developments, we would like to explore in this *commentary and concept* article whether a more adaptive, dynamic algorithm could improve patient outcomes.

## History of CPR cycle in AHA and ERC adult BLS guidelines

In 1998, rhythm analysis was performed every minute if the rhythm was shockable, or every three minutes if the rhythm was non-shockable.^4^ The guidelines were modified in 2000 to require an analysis every minute for all rhythms (shockable/non-shockable).^5^ The intention of this simplification was to aid the acquisition and retention of knowledge and skills.

Five years later, in 2005, several treatment recommendations were changed simultaneously to increase the CCF. These changes included going from three stack shocks in case of persistent VF to only one; the pulse check, which was performed in case of non-shockable rhythm, was removed; and the ratio of CC to ventilations was changed to 30:2 in adults.

For CPR duration, the International Liaison Committee on Resuscitation (ILCOR) recommended performing five cycles of CPR between each rhythm analysis.^6^ Consequently, the European Resuscitation Council (ERC) recommended CPR for two minutes between each analysis, as five cycles of 30:2-CPR take almost two minutes and rescuers found it difficult to count five cycles of CPR.^3^ It was also considered easier to train people to perform 30:2-CPR for a set period of time rather than a specific number of cycles. Subsequently, ILCOR has examined the evidence for alternate durations, but as detailed below, the only studies found were conducted with older CPR algorithms. However, two recent observational studies do challenge this point suggesting from one minute to more than four minutes CPR in function of rhythm (shockable/non-shockable).^7^, ^8^ This topic relates to two ILCOR systematic reviews: “BLS-2211-Rhythm Analysis during Compressions” and “BLS-2212-Duration of CPR cycles”.^9^, ^10^

The changes in the 1998 to 2020-guidelines and their implications for the analysis/CPR cycle are summarised in Fig. 1. We estimated theoretical CCF and potential time in VF (VF duration) for each set of guidelines, with detailed calculations (Supplementary file). In 2005, the estimated CCF rose to 71–72 %, but the estimated VF duration also increased to 67 s. Moreover, Berdowski *et al.* found that the median times in initial VF and in recurrent VF were significantly higher with the 2005-guidelines than with the 2000-guidelines.^11^

## Why a fixed two-minute interval?

Out-of-hospital cardiac arrest (OHCA) resuscitation strategies have evolved significantly over the past decades, with a primary focus on optimizing CPR quality and minimizing interruptions. The 2020-algorithm mandates a 2-minute cycle of CC and ventilation followed by rhythm analysis and potential defibrillation.

### Why a pause for analysis?

Chest compressions generate significant artifacts on the electrocardiogram (ECG), making it difficult for an automated external defibrillator (AED) to analyse the rhythm accurately. Consequently, a pause in CPR was necessary for reliable rhythm analysis.

### Why a fixed interval between analysis pause?

As a pause in CPR is needed, analysing rhythm too frequently reduces the CCF, potentially harming the patient by limiting the flow of oxygenated blood to vital organs. Conversely, if the patient has a shockable rhythm, prolonged CPR could delay defibrillation, increasing the risk of asystole.^11^ An optimal care would be to have a short interval between analysis pause when the rhythm is shockable, to prioritize the defibrillation, and a long interval when the rhythm is non-shockable to maximize CCF. That was the case in 1998.^4^ However, a fixed interval facilitated the work of the rescuers who thus became more efficient.

### Why two minutes?

The 2-minute interval has remained an ILCOR recommendation for 20 years.^12^, ^13^ However, the optimal duration is still unknown. The two most recent ILCOR reviews cite two randomized controlled trials (RCT) in support of this recommendation, while acknowledging that the evidence remains weak.^14^, ^10^ Higher value was placed on maintaining consistency with previous recommendations.

The first RCT, from 2003, compared a protocol of one minute of 15:2-CPR for shockable rhythms and three minutes for non-shockable rhythms (control) with a protocol of three minutes for all rhythms (intervention). None of the patients received 2-minute CPR, and this study found no benefit from the intervention compared to the control for any of the studied outcomes.^15^

In the second RCT, dated 2008, the first group consisted of patients who were treated after the implementation of the 2005-guidelines, which introduced single shocks, 30:2-CPR, and 2-minute CPR cycles. The second group consisted of patients who had been treated before the implementation of these guidelines and had therefore received stacked shocks (up to three in cases of persistent VF/VT), 15:2-CPR, and 1-minute CPR cycles. No clear benefit was observed for either guideline.^16^

Despite the widespread adoption of this protocol, there is no definitive evidence that two minutes represents the optimal interval for all cases. Adherence to a rigid 2-minute protocol may not align with the variability observed in clinical resuscitation scenarios, where patient-specific factors can significantly influence the optimal timing of interventions.^17^

## The advancements in technology

Since the implementation of the 2-minute CPR algorithm in 2005, significant advancements have been made in defibrillation technology. A seemingly small but impactful change is that the AED devices tell rescuers when to continue and stop CPR. Rescuers are not actually waiting two minutes before stopping CPR, they are waiting for instructions from the AED.

A recent significant evolution is the capability for rhythm analysis during CC. Indeed, the improvements in the size and quality of real life OHCA databases have enabled machine learning models to enhance the accuracy of ECG rhythm detection. Moreover, signal processing techniques have also evolved, enabling more precise noise reduction and feature extraction, which further refines the interpretation of cardiac rhythms.

Recently, many manufacturers have developed new detectors capable of distinguishing ECG rhythms during active CC.^18^, ^19^, ^20^, ^21^, ^22^, ^23^, ^24^, ^25^ Some detectors were clinically tested on the field by de Graaf *et al.* and Derkenne *et al.* with good sensitivity and specificity (Table 1).^7^, ^8^ The latest ILCOR review weakly recommends evaluating the usefulness of ECG rhythm analysis during CPR through clinical trials or research initiatives.^9^

**Table 1:** Sensitivity and specificity of rhythm analysis during compression compared to American Heart Association performance goals.

	**De Graaf *et al.*** ^7^	**Derkenne *et al.*** ^8^	**AHA Goals** ^34^
**VF (sensitivity)**	95.4 %	91.4 %	> 90 %
**Asystole (specificity)**	97.5 %	100 %	>95 %
**Other non-shockable (specificity)**	99.5 %	99.7 %	>95 %

VF: Ventricular Fibrillation, AHA: American Heart Association.

The development of new shock-advisory systems could enabled continuous rhythm analysis during CC, potentially negating the need for routine pauses in CPR. As Roman-Pognuz and Ristagno argued in their editorial, to shock or not to shock is no longer a question, new AED should be able to maximize CCF and minimize time to defibrillation.^26^

## A future algorithm?

**Fig. 2:**
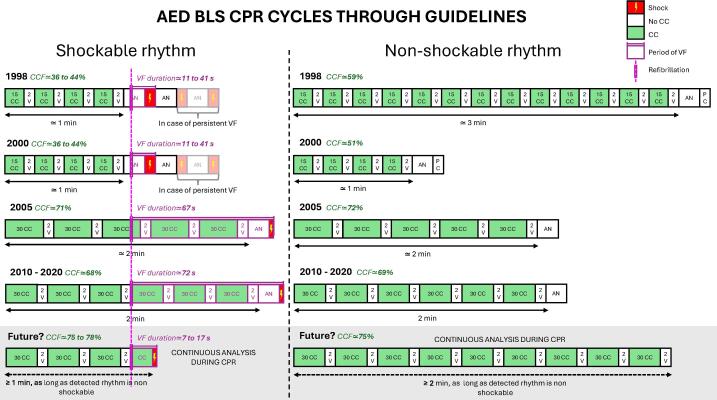
Representation of the typical analysis/CPR cycles in steady state, for shockable and non-shockable rhythms, considering the changes in adult basic life support guidelines (AHA/ERC), with a proposition of target cycles for future. Chest compression fraction and ventricular fibrillation duration are estimated in Additional file with a time to refibrillation set to 1 min. Refibrillation appearing less than 5 s after a shock is defined as persistent ventricular fibrillation. AED: Automated External Defibrillator; AHA: American Heart Association; BLS: Basic Life Support; AN: Analysis; CC: Chest Compression; CCF: Chest Compression Fraction; CPR: Cardiopulmonary resuscitation; ERC: European Resuscitation Council; PC: Pulse Check; V: Ventilation; VF: Ventricular Fibrillation.

Given these technological advancements, a new adaptative analysis/CPR algorithm could be envisioned. A proposed alternative approach would be to integrate continuous rhythm analysis into CPR procedures, allowing for immediate defibrillation in cases of detected VF/VT without waiting for the next scheduled analysis. Such an approach would capitalize on recent technological advancements while ensuring that CC remain uninterrupted in non-shockable rhythms. A potential future algorithm in steady state (i.e. after the first analysis) is presented and compared to previous ones (Fig. 2).

Nevertheless, implementing such a change would require retraining of emergency medical personnel and reconfigurations of AED programming. While these are valid logistical challenges, they must be weighed against the potential for significantly improved patient outcomes. If a real-time, adaptive rhythm analysis approach leads to higher survival rates and better neurological recovery, the benefits would likely outweigh the transitional difficulties. The question then arises: is it worth revising the algorithm?

A two-step process could be applied for the algorithm revision: before the continuous rhythm analysis is widespread, it could be astute to broaden the CPR duration as it was advised in 1998.

## Advantages for shockable rhythms

Telesz *et al.* reported in their study a median time to refibrillation (recurrences of VF) of 25 s [interquartile range: 11–66].^27^ Those results were confirmed by Hong Tuan Ha *et al.* in their letter to the editor, they found that 82 % of refibrillations occur within the first minute post-shock.^28^ Those results raise a fundamental concern: if most VF patients refibrillate within 60 s, then waiting an additional minute before the shock may result in prolonged time spent in VF. We estimate that with a continuous analysis during CPR, the VF duration per cycle would be between 7 and 17 s, whereas it is 72 s with the 2020-guidelines (Supplementary file).

Clinical evidence supports the idea that rhythm analysis during CC leads to faster shocks for patients in VF. A study from 2024 showed that rhythm analysis during CPR resulted in earlier shocks for 91.4 % of VF patients.^29^ Additionally, Derkenne *et al.* found that the median time between refibrillation and the next shock was reduced by 64 s.^8^ Moreover, de Graaf *et al.* found that with continuous rhythm monitoring, the median pre-shock pause was shorter compared to control cases (8 s vs 22 s, *p* < 0.001).^7^

A study by Awad *et al.* investigated the correlation between intra-arrest VF/VT duration and return of spontaneous circulation (ROSC) in OHCA patients.^30^ The findings indicated that longer durations of VF/VT significantly reduced the likelihood of ROSC. Additionally, Eilevstjønn *et al.* found that median VF duration was shorter in shocks producing ROSC.^31^

VF is a high-energy demanding dysrhythmia and earlier termination may lead to better outcomes with an organized rhythm and possibly a pulse and even survival. Since prolonged time in VF deteriorates cardiac function and increases the likelihood of asystole, minimizing the duration of VF should improve survival rates.^11^ In the study of Derkenne *et al.*, survival improved within a subgroup of patients who had OHCA in public places and within a short time between call to AED connection.^8^.

## Advantages for non-shockable rhythms

For patients in non-shockable rhythms, maximizing CCF is crucial. Eliminating fixed timing of rhythm analysis reduces unnecessary pauses, thus increasing total CPR time and improving perfusion. We estimate that if analysis during CPR allows CPR to continue without interruption, the CCF would be 75 % (30:2, 5 s for ventilation, Supplementary file). However, it may require re-evaluating the standard intervals for switching compressors to prevent fatigue; such transitions could be performed during ventilations to avoid introducing additional pauses.

A study showed that rhythm analysis during CPR resulted in uninterrupted CPR 86.5 % of the time for asystole and 95.2 % for other non-shockable rhythms.^29^ Interventions with continuous rhythm monitoring had significantly higher median CCF (86 % vs 80 %, *p* < 0.001) in the study of de Graaf et al.^7^

Moreover, three separate retrospective studies have suggested that modifying CPR algorithms to shorten pre-shock pauses for VF patients and avoid unnecessary interruptions for non-VF patients could enhance survival rates.^7^, ^32^, ^33^

Given the strong correlation between uninterrupted CPR and positive outcomes, a new algorithm leveraging continuous rhythm analysis under CC may be the next step in CPR evolution.

## Conclusions

Traditionally, rhythm analysis required interruptions in CC to obtain clear ECG readings. However, technological advancements have enabled rhythm analysis to be conducted during ongoing CC.

Recent studies indicate that refibrillation often occurs within one minute post-shock, suggesting that prolonged CPR cycles before reanalysis may inadvertently delay defibrillation, ultimately reducing ROSC rates. On the other hand, patients with non-shockable rhythm would benefit from a longer CPR period.

While the standardized interval simplified training and implementation, a more flexible and personalised, real-time approach could provide superior clinical outcomes.

With the advent of continuous ECG analysis and improved AED technology, the debate is no longer about whether to shock but when to shock optimally. The extent to which the 2-minute algorithm needs to be changed remains uncertain, but it is clear that a reassessment is necessary.

## CRediT authorship contribution statement

**Emma Menant:** Writing – original draft, Visualization, Investigation, Conceptualization. **Guillaume Debaty:** Writing – review & editing. **Janet Bray:** Writing – review & editing. **Thomas Rea:** Writing – review & editing. **Xavier Jouven:** Writing – review & editing, Supervision, Project administration, Conceptualization.

## Declaration of competing interest

The authors declare the following financial interests/personal relationships which may be considered as potential competing interests: Emma Menant reports a relationship with SCHILLER Médical, Wissembourg, France that includes: receiving research support. Xavier Jouven reports a relationship with SCHILLER Médical, Wissembourg, France that includes: receiving funds for scientific consulting. Thomas Rea reports a relationship with Philips Healthcare that includes: funding grants. ECG analysis during chest compressions is both timely and critically important. Therefore, major AED manufacturers—including Schiller and Philips—are actively addressing this challenge through various approaches. However, it should be noted that Schiller and Philips funding supports had no influence in the conception, writing, or review of this scientific paper. Furthermore, the authors did not receive any financial or in-kind support for the writing of this article. Given their role as associate editor, Janet Bray had no involvement in the peer-review of this article and has no access to information regarding its peer-review. Full responsibility for the editorial process for this article was delegated to another journal editor. If there are other authors, they declare that they have no known competing financial interests or personal relationships that could have appeared to influence the work reported in this paper.
