# Supplementary material for: Rethinking the 2-minute rule in adult basic life support cardiopulmonary resuscitation
Source: Resusc Plus. 2025 Aug 23;26:101070. doi: 10.1016/j.resplu.2025.101070 (PMC12441612; doi:10.1016/j.resplu.2025.101070)
Supplement: Supplementary Data 1 [file mmc1.docx]

**Supplementary Material**

**Rethinking the 2-minute rule in adult basic life support cardiopulmonary resuscitation**

Emma Menant^a b^*, Guillaume Debaty^c d^, Janet Bray^e f^, Thomas Rea^g h^, Xavier Jouven^a b^

Affiliations:

^a^ Université Paris Cité, Inserm, PARCC, F-75015 Paris, France

^b^ Paris Cardiac Arrest Center, European Georges Pompidou Hospital, APHP, Paris, France

^c^ Emergency Department and Mobile Intensive Care Unit, University Hospital of Grenoble Alpes, Grenoble, France

^d^ Univ. Grenoble Alpes, CNRS, UMR 5525, VetAgro Sup, Grenoble INP, TIMC, 38000 Grenoble, France

^e^ School of Public Health and Preventive Medicine, Monash University, Melbourne, Australia

^f^ Prehospital, Resuscitation and Emergency Care Research Unit (PRECRU), School of Nursing, Midwifery and Paramedicine, Curtin University, Bentley, Australia

^g^ Department of Medicine, University of Washington, Seattle, Washington, United States

^h^ King County Emergency Medical Services Seattle-King County Department of Public Health Seattle WA United States

**Estimation of chest compression fraction and ventricular fibrillation duration during adult basic life support cardiopulmonary resuscitation cycles through guidelines**

**Glossary**

| ABC | Airway-Breathing-Compression |
| --- | --- |
| AN | Analysis |
| BLS | Basic Life Support |
| CAB | Compression-Airway-Breathing |
| CC | Chest Compression |
| CCF | Chest Compression Fraction |
| CPR | Cardiopulmonary Resuscitation |
| OHCA | Out-of-hospital cardiac arrest |
| PC | Pulse Check |
| V | Ventilation |
| VF | Ventricular Fibrillation |

1. **Estimation**

A cycle refers to the smallest sequence of events that regularly repeats in the same order during resuscitation. It implies that cardiopulmonary resuscitation (CPR) has reached a steady state (i.e. after the first analysis), meaning that compressions and ventilations follow a consistent rhythm with minimal disruptions. This contrasts with the initial phase, which follows its own guidelines—such as an initial analysis outside CPR and an ABC or CAB order depending on the year—as well as adjustments or interruptions due to the installation of basic life support (BLS) rescuers.

A cycle is composed of different components that may vary between shockable and non-shockable rhythms or over the years. These components include analysis, pulse check, shock, ventilation and chest compressions (CC). The duration of each component can vary significantly between interventions. Fixed estimated component durations were chosen to facilitate the calculation and comparison across years. All estimated component durations for adult out-of-hospital cardiac arrest (OHCA) are summarised in Table 1.

We assume that an analysis during a CPR pause takes 10 seconds. [1], [2], [3]

We assume that pulse check takes 5 seconds. [4]

We assume that before 2005, a shock took 5 seconds because the capacitors were not pre-charged, and that since 2005, it has taken 2 seconds because the capacitors are pre-charged, allowing the shock to be delivered immediately after the AED gives the instruction to shock. [5]

We assume that two ventilations take 5 seconds. [6], [7], [8]

Performing 15 CC at a rate of 100/min takes 9 seconds, 30 CC at 100/min takes 18 seconds and 30 CC at 120/min takes 15 seconds.

We choose a time to refibrillation of 60 seconds. [9], [10]

We assume that time to VF detection during CPR should take 5 to 15 seconds. The current algorithm are around 15 seconds. [11], [12], [13], [14]

| **Cycle component** | **Estimated duration** |
| --- | --- |
| Analysis | $\boldsymbol{a}=\boldsymbol{10} s$ |
| Pulse check | $\boldsymbol{pc}=\boldsymbol{5} s$ |
| Shock 1998/2000 (without pre-charge) | $\boldsymbol{s}\boldsymbol{1}=\boldsymbol{5} s$ |
| Shock 2005+ (with pre-charge) | $\boldsymbol{s}\boldsymbol{2}=\boldsymbol{2} s$ |
| 2 Ventilations | $\boldsymbol{v}=\boldsymbol{5} s$ |
| 15 CC at 100/min | $\boldsymbol{c}\boldsymbol{1}=15*\frac{60}{100}=\boldsymbol{9} s$ |
| 30 CC at 100/min | $\boldsymbol{c}\boldsymbol{2}=30*\frac{60}{100}=\boldsymbol{18} s$ |
| 30 CC at 120/min | $\boldsymbol{c}\boldsymbol{3}=30*\frac{60}{120}=\boldsymbol{15} s$ |
| Time to refibrillation | $\boldsymbol{r}=\boldsymbol{60} s$ |
| Time to VF detection during CPR | $\boldsymbol{d}\in[\boldsymbol{5}-\boldsymbol{15}\boldsymbol{]} s$ |

Table 1 : Estimation of cycle component durations

1. **Method**
   1. ***Chest Compression Fraction***

The chest compression fraction (CCF) is the proportion of the resuscitation cycle during which CC are performed.

$$CCF=\frac{CC duration}{Cycle duration}$$

Equation 1 : Chest compression fraction

- 1. ***CPR number***

CPR number is the number of CC period during the cycle. It corresponds to the number of CC period followed by ventilations (**c** + **v**) that can be done in the CPR duration.

As CPR duration do not always correspond to an entire number of CC followed by ventilations (**c** + **v**) period, we choose the CPR number that is closest to the CPR duration.

Actually, AED are asking the rescuer to stop CPR after an entire number of CC and ventilation period and not exactly after 1, 2 or 3 minutes.

*Example:*


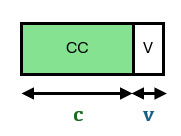


Figure 1 : Representation of one CC and ventilation period

The required number of CC is 15 at a rate of 100/min, taking 9 seconds.

$$\boldsymbol{c}=15*\frac{60}{100}=9 s$$

CC are followed by 2 ventilations, taking 5 seconds.

$$\boldsymbol{c}+\boldsymbol{v}=9+5=14 s$$

- Required CPR duration is 1 minute.

$$4*\left( \boldsymbol{c}+\boldsymbol{v} \right)=56 s\approx60 s$$

$$CPR number=4$$

- Required CPR duration is 3 minutes.

$$13*\left( \boldsymbol{c}+\boldsymbol{v} \right)=182 s\approx180 s$$

$$CPR number=13$$

- 1. ***CC duration***

CC duration is time spent doing chest compressions during the cycle.

$$CC duration=CPR number*CC period duration$$

Equation 2 : Chest compression duration

CC period duration is **9** seconds in 1998/2000, **18** seconds in 2005 and **15** seconds in 2010/2015/2020 (Table 1).

- 1. ***VF duration***

VF duration refers to the time spent in ventricular fibrillation during one cycle. A VF period begins with a refibrillation and ends with an effective shock. For this estimation, we assume that refibrillation occurs one minute after the previous shock.

$$VF duration=Time bewteen two effective shocks-Time to refibrillation$$

Equation 3 : Ventricular fibrillation duration


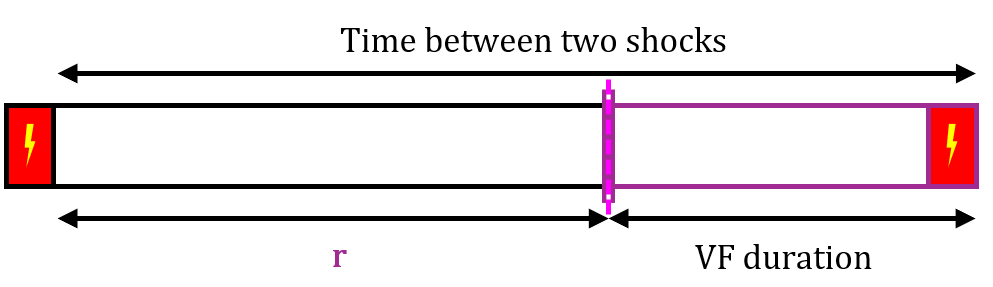


Figure 2 : Representation of ventricular fibrillation duration

1. **1998**
   1. ***Non-Shockable***

In 1998, for adult non-shockable rhythm, analysis was followed by pulse check. Then, 3 minutes of CPR was performed. CPR was composed of 15 CC followed by 2 ventilations and CC was performed at a rate of 100/min. Figure 3 is a representation of a cycle.

**
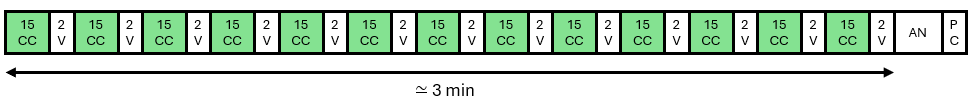
**

Figure 3 : Representation of the typical analysis/CPR cycle in steady state, for non-shockable rhythms, considering the 1998 guidelines. AN: Analysis; CC: Chest Compression; V: Ventilation; PC: Pulse Check

**The estimated CCF for non-shockable rhythm in 1998 is 59 %** (see Equation 4 below).

$$13*\left( \boldsymbol{c}\boldsymbol{1}+\boldsymbol{v} \right)=182 s\approx3*60 s$$

$$CPR number=13$$

$$CC duration=CPR number*\boldsymbol{c}\boldsymbol{1}=13*\boldsymbol{9}=117 s$$

$$Cycle duration\boldsymbol{=}CPR number*\left( \boldsymbol{c}\boldsymbol{1}+\boldsymbol{v} \right)+\boldsymbol{a}+\boldsymbol{pc}=182+\boldsymbol{10}+\boldsymbol{5}=197 s$$

$$CCF=\frac{CC duration}{Cycle duration}=\frac{117}{197}=59 \%$$

Equation 4 : Detailed calculation of chest compression fraction for non-shockable rhythm considering the 1998 guidelines

- 1. ***Shockable***

For adult shockable rhythm, the analysis was followed by a shock. Then, a second analysis was performed. If the rhythm was non-shockable, CPR was resumed (Figure 4). If the rhythm was still shockable (persistent VF), a second shock was administered and a third analysis was done. If the rhythm was still shockable a final and third shock was administered and CPR was resumed (Figure 5), else CPR was resumed.

1 minute of CPR was performed. CPR was composed of 15 CC followed by 2 ventilations. The CC was performed at a rate of 100/min.


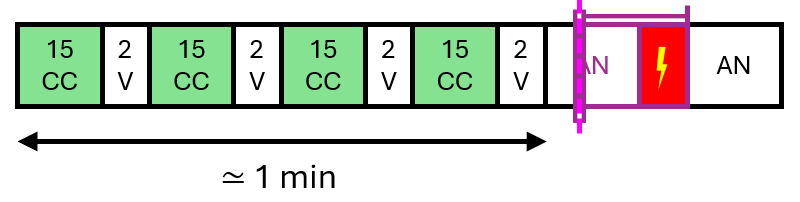


Figure 4 : Representation of the typical analysis/CPR cycle in steady state, for shockable rhythms, without persistent ventricular fibrillation, considering the 1998 guidelines. AN: Analysis; CC: Chest Compression; V: Ventilation


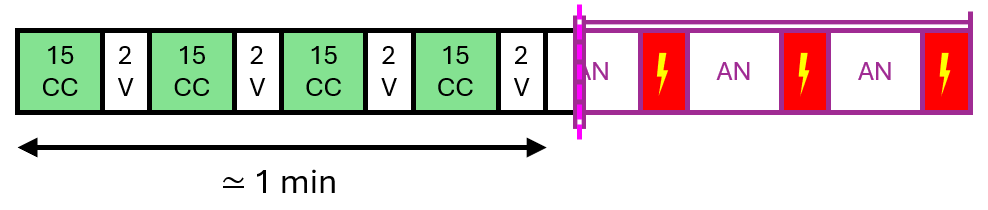


Figure 5 : Representation of the typical analysis/CPR cycle in steady state, for shockable rhythms, with persistent ventricular fibrillation, considering the 1998 guidelines. AN: Analysis; CC: Chest Compression; V: Ventilation

**The estimated CCF for shockable rhythms in 1998 is between 36 and 44 %** (see Equation 5 below).

$$4*\left( \boldsymbol{c}\boldsymbol{1}+\boldsymbol{v} \right)=56 s\approx60 s$$

$$CPR number=4$$

$$CC duration=CPR number*\boldsymbol{c}\boldsymbol{1}=4*\boldsymbol{9}=36 s$$

If 1 shock (Figure 4):

$${Cycle duration}_{min}\boldsymbol{=}CPR number*\left( \boldsymbol{c}\boldsymbol{1}+\boldsymbol{v} \right)+\boldsymbol{a}+\boldsymbol{s}\boldsymbol{1}+\boldsymbol{a}$$

$${Cycle duration}_{min}=56+\boldsymbol{10}+\boldsymbol{5}+\boldsymbol{10}=81 s$$

$${CCF}_{max}=\frac{CC duration}{{Cycle duration}_{min}}=\frac{36}{81}=0.44$$

If 3 shocks (Figure 5):

$${Cycle duration}_{max}=CPR number*\left( \boldsymbol{c}\boldsymbol{1}+\boldsymbol{v} \right)+3*\left( \boldsymbol{a}+\boldsymbol{s}\boldsymbol{1} \right)$$

$${Cycle duration}_{max}=56+3*\left( \boldsymbol{10}+\boldsymbol{5} \right)=101 s$$

$${CCF}_{min}=\frac{CC duration}{{Cycle duration}_{max}}=\frac{36}{101}=0.36$$

$$CCF\in\left[ 36-44 \right] \%$$

Equation 5 : Detailed calculation of chest compression fraction for shockable rhythm considering the 1998 guidelines

**The estimated VF duration for shockable rhythm in 1998 is between 11 and 41 s** (see Equation 6 below).

If 1 shock (Figure 4):

$${Time between two effective shocks}_{min}=CPR number*\left( \boldsymbol{c}\boldsymbol{1}+\boldsymbol{v} \right)+\boldsymbol{a}+\boldsymbol{s}\boldsymbol{1}$$

$${Time between two effective shocks}_{min}=56+\boldsymbol{10}+\boldsymbol{5}=71 s$$

$${VF duration}_{min}={Time between two effective shocks}_{min}-\boldsymbol{r}=71-\boldsymbol{60}=11 s$$

If 3 shocks (Figure 5):

$${Time between two effective shocks}_{max}=CPR number*\left( \boldsymbol{c}\boldsymbol{1}+\boldsymbol{v} \right)+3*(\boldsymbol{a}+\boldsymbol{s}\boldsymbol{1}\boldsymbol{)}$$

$${Time between two effective shocks}_{max}=56+3*(\boldsymbol{10}+\boldsymbol{5}\boldsymbol{)}=101 s$$

$${VF duration}_{max}={Time between two effective shocks}_{max}-\boldsymbol{r}=101-\boldsymbol{60}=41 s$$

$$CCF\in\left[ 11-41 \right] s$$

Equation 6 : Detailed calculation of ventricular fibrillation duration for shockable rhythm considering the 1998 guidelines

1. **2000**
   1. ***Non-Shockable***

In 2000, for adult non-shockable rhythm, the analysis was followed by pulse check. Then, 1 minute of CPR was performed. CPR was composed of 15 CC followed by 2 ventilations and CC was performed at a rate of 100/min. Figure 6 is a representation of a cycle.

**
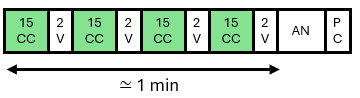
**

Figure 6 : Representation of the typical analysis/CPR cycle in steady state, for non-shockable rhythms, considering the 2000 guidelines. AN: Analysis; CC: Chest Compression; V: Ventilation; PC: Pulse Check

**The estimated CCF for non-shockable rhythm in 2000 is 51 %** (see Equation 7 below).

$$4*\left( \boldsymbol{c}\boldsymbol{1}+\boldsymbol{v} \right)=56 s\approx60 s$$

$$CPR number=4$$

$$CC duration=CPR number*\boldsymbol{c}\boldsymbol{1}=4*\boldsymbol{9}=36 s$$

$$Cycle duration\boldsymbol{=}CPR number*\left( \boldsymbol{c}\boldsymbol{1}+\boldsymbol{v} \right)+\boldsymbol{a}+\boldsymbol{pc}=56+\boldsymbol{10}+\boldsymbol{5}=71 s$$

$$CCF=\frac{CC duration}{Cycle duration}=\frac{36}{71}=51 \%$$

Equation 7 : Detailed calculation of chest compression fraction for non-shockable rhythm considering the 2000 guidelines

- 1. ***Shockable***

For adult shockable rhythm, the analysis was followed by a shock. Then, a second analysis was performed. If the rhythm was non-shockable, CPR was resumed (Figure 7). If the rhythm was still shockable (persistent VF), a second shock was administered and a third analysis was done. If the rhythm was still shockable a final and third shock was administered and CPR was resumed (Figure 8), else CPR was resumed.

1 minute of CPR was performed. CPR was composed of 15 CC followed by 2 ventilations. The CC was performed at a rate of 100/min.


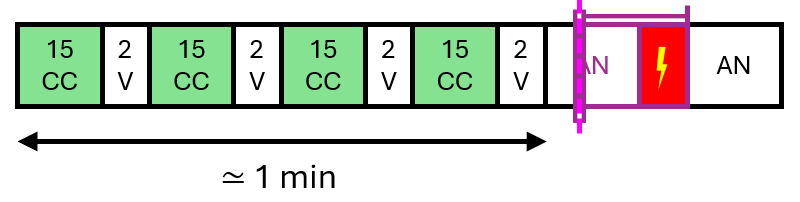


Figure 7 : Representation of the typical analysis/CPR cycle in steady state, for shockable rhythms, without persistent ventricular fibrillation, considering the 2000 guidelines. AN: Analysis; CC: Chest Compression; V: Ventilation


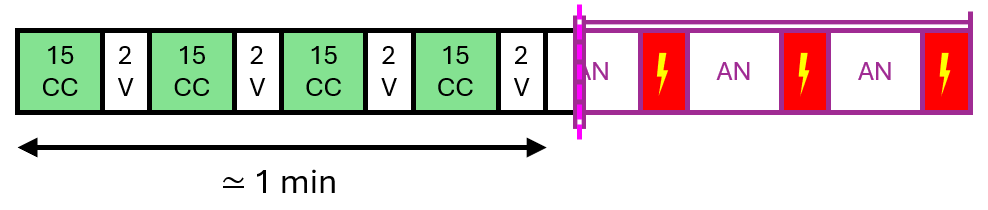


Figure 8 : Representation of the typical analysis/CPR cycle in steady state, for shockable rhythms, with persistent ventricular fibrillation, considering the 2000 guidelines. AN: Analysis; CC: Chest Compression; V: Ventilation

**The estimated CCF for shockable rhythm in 2000 is between 36 and 44 %** (see Equation 8 below).

$$4*\left( \boldsymbol{c}\boldsymbol{1}+\boldsymbol{v} \right)=56 s\approx60 s$$

$$CPR number=4$$

$$CC duration=CPR number*\boldsymbol{c}\boldsymbol{1}=4*\boldsymbol{9}=36 s$$

If 1 shock (Figure 7):

$${Cycle duration}_{min}\boldsymbol{=}CPR number*\left( \boldsymbol{c}\boldsymbol{1}+\boldsymbol{v} \right)+\boldsymbol{a}+\boldsymbol{s}\boldsymbol{1}+\boldsymbol{a}=56+\boldsymbol{10}+\boldsymbol{5}+\boldsymbol{10}=81 s$$

$${CCF}_{max}=\frac{CC duration}{{Cycle duration}_{min}}=\frac{36}{81}=0.44$$

If 3 shocks (Figure 8):

$${Cycle duration}_{max}=CPR number*\left( \boldsymbol{c}\boldsymbol{1}+\boldsymbol{v} \right)+3*\left( \boldsymbol{a}+\boldsymbol{s}\boldsymbol{1} \right)$$

$${Cycle duration}_{max}=56+3*\left( \boldsymbol{10}+\boldsymbol{5} \right)=101 s$$

$${CCF}_{min}=\frac{CC duration}{{Cycle duration}_{max}}=\frac{36}{101}=0.36$$

$$CCF\in\left[ 36-44 \right] \%$$

Equation 8 : Detailed calculation of ventricular fibrillation duration for shockable rhythm considering the 2000 guidelines

**The estimated VF duration for shockable rhythm in 2000 is 11 to 41 s** (see Equation 9 below).

If 1 shock (Figure 7):

$${Time between two effective shocks}_{min}=CPR number*\left( \boldsymbol{c}\boldsymbol{1}+\boldsymbol{v} \right)+\boldsymbol{a}+\boldsymbol{s}\boldsymbol{1}$$

$${Time between two effective shocks}_{min}=56+\boldsymbol{10}+\boldsymbol{5}=71 s$$

$${VF duration}_{min}={Time between two effective shocks}_{min}-\boldsymbol{r}=71-\boldsymbol{60}=11 s$$

If 3 shocks (Figure 8):

$${Time between two effective shocks}_{max}=CPR number*\left( \boldsymbol{c}\boldsymbol{1}+\boldsymbol{v} \right)+3*(\boldsymbol{a}+\boldsymbol{s}\boldsymbol{1}\boldsymbol{)}$$

$${Time between two effective shocks}_{max}=56+3*(\boldsymbol{10}+\boldsymbol{5}\boldsymbol{)}=101 s$$

$${VF duration}_{max}={Time between two effective shocks}_{max}-\boldsymbol{r}=101-\boldsymbol{60}=41 s$$

$$VF duration\in\left[ 11-41 \right] s$$

Equation 9 : Detailed calculation of ventricular fibrillation duration for shockable rhythm considering the 2000 guidelines

1. **2005**
   1. ***Non-Shockable***

In 2005, for adult non-shockable rhythm, the analysis was followed by 2 minutes of CPR. CPR was composed of 30 CC followed by 2 ventilations and CC was performed at a rate of 100/min. Figure 9 is a representation of a cycle.

**
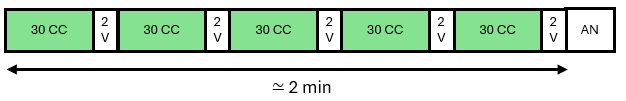
**

Figure 9 : Representation of the typical analysis/CPR cycle in steady state, for non-shockable rhythms, considering the 2005 guidelines. AN: Analysis; CC: Chest Compression; V: Ventilation

**The estimated CCF for non-shockable rhythm in 2005 is 72 %** (see Equation 10 below).

$$5*\left( \boldsymbol{c}\boldsymbol{2}+\boldsymbol{v} \right)=115 s\approx2*60 s$$

$$CPR number=5$$

$$CC duration=CPR number*\boldsymbol{c}\boldsymbol{2}=5*\boldsymbol{18}=90 s$$

$$Cycle duration\boldsymbol{=}CPR number*\left( \boldsymbol{c}\boldsymbol{2}+\boldsymbol{v} \right)+\boldsymbol{a}=115+\boldsymbol{10}=125 s$$

$$CCF=\frac{CC duration}{Cycle duration}=\frac{90}{125}=72 \%$$

Equation 10 : Detailed calculation of chest compression fraction for non-shockable rhythm considering the 2005 guidelines

- 1. ***Shockable***

For adult shockable rhythm, the analysis was followed by a shock. Then, 2 minutes of CPR was performed. CPR was composed of 30 CC followed by 2 ventilations. CC was performed at a rate of 100/min. Figure 10 is a representation of a cycle.

**
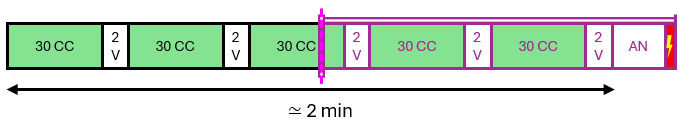
**

Figure 10 : Representation of the typical analysis/CPR cycle in steady state, for shockable rhythms, considering the 2005 guidelines. AN: Analysis; CC: Chest Compression; V: Ventilation

**The estimated CCF for shockable rhythm in 2005 is 71 %** (see Equation 11 below).

$$5*\left( \boldsymbol{c}\boldsymbol{2}+\boldsymbol{v} \right)=115 s\approx2*60 s$$

$$CPR number=5$$

$$CC duration=CPR number*\boldsymbol{c}\boldsymbol{2}=5*\boldsymbol{18}=90 s$$

$$Cycle duration\boldsymbol{=}CPR number*\left( \boldsymbol{c}\boldsymbol{2}+\boldsymbol{v} \right)+\boldsymbol{a}+\boldsymbol{s}\boldsymbol{2}=115+\boldsymbol{10}+\boldsymbol{2}=127 s$$

$$CCF=\frac{CC duration}{Cycle duration}=\frac{90}{127}=71 \%$$

Equation 11 : Detailed calculation of ventricular fibrillation duration for shockable rhythm considering the 2005 guidelines

**The estimated VF duration for shockable rhythm in 2005 is 67 s** (see Equation 12 below).

$$Interval between two shocks=CPR number*\left( \boldsymbol{c}\boldsymbol{2}+\boldsymbol{v} \right)+\boldsymbol{a}+\boldsymbol{s}\boldsymbol{2}=115+\boldsymbol{10}+\boldsymbol{2}=127 s$$

$$VF duration=Interval bewteen two shocks-\boldsymbol{r}=127-\boldsymbol{60}=67 s$$

Equation 12 : Detailed calculation of ventricular fibrillation duration for shockable rhythm considering the 2005 guidelines

1. **2010 -2020**
   1. ***Non-Shockable***

In 2010-2020 adult guidelines, the recommended procedure for non-shockable rhythm, was to resume CPR after the analysis. CPR was composed of 30 CC followed by 2 ventilations for 2 minutes. The CC was performed at a rate of 120/min. Figure 11 is a representation of a cycle.

**
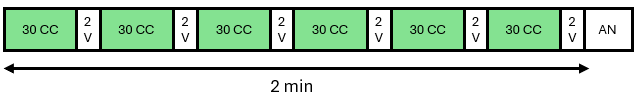
**

Figure 11 : Representation of the typical analysis/CPR cycle in steady state, for non-shockable rhythms, considering the 2010-2020 guidelines. AN: Analysis; CC: Chest Compression; V: Ventilation

**The estimated CCF for non-shockable rhythm from 2010 to 2020 is 69 %** (see Equation 13 below).

$$6*\left( \boldsymbol{c}\boldsymbol{3}+\boldsymbol{v} \right)=120 s=2*60 s$$

$$CPR number=6$$

$$CC duration=CPR number*\boldsymbol{c}\boldsymbol{3}=6*\boldsymbol{15}=90 s$$

$$Cycle duration\boldsymbol{=}CPR number*\left( \boldsymbol{c}\boldsymbol{3}+\boldsymbol{v} \right)+\boldsymbol{a}=120+\boldsymbol{10}=130 s$$

$$CCF=\frac{CC duration}{Cycle duration}=\frac{90}{130}=69 \%$$

Equation 13 : Detailed calculation of chest compression fraction for non-shockable rhythm considering the 2010-2020 guidelines

- 1. ***Shockable***

The recommended procedure for shockable rhythm, was to shock after the analysis. Then, CPR was resumed for 2 minutes. It was composed of 30 CC followed by 2 ventilations and CC was performed at a rate of 120/min. Figure 12 is a representation of a cycle.

**
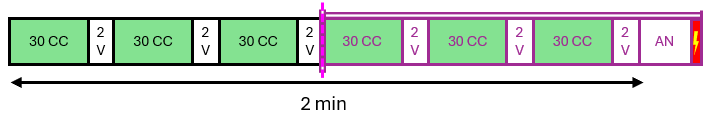
**

Figure 12 : Representation of the typical analysis/CPR cycle in steady state, for shockable rhythms, considering the 2010-2020 guidelines. AN: Analysis; CC: Chest Compression; V: Ventilation

**The estimated CCF for shockable rhythm from 2010 to 2020 is 68 %** (see Equation 14 below).

$$6*\left( \boldsymbol{c}\boldsymbol{3}+\boldsymbol{v} \right)=120 s=2*60 s$$

$$CPR number=6$$

$$CC duration=CPR number*\boldsymbol{c}\boldsymbol{3}=6*\boldsymbol{15}=90 s$$

$$Cycle duration\boldsymbol{=}CPR number*\left( \boldsymbol{c}\boldsymbol{3}+\boldsymbol{v} \right)+\boldsymbol{a}+\boldsymbol{s}\boldsymbol{2}=120+\boldsymbol{10}+\boldsymbol{2}=132 s$$

$$CCF=\frac{CC duration}{Cycle duration}=\frac{90}{132}=68 \%$$

Equation 14 : Detailed calculation of ventricular fibrillation duration for shockable rhythm considering the 2010-2020 guidelines

**The estimated VF duration for shockable rhythm from 2010 to 2020 is 72 s** (see Equation 15 below).

$$Interval between two shocks=CPR number*\left( \boldsymbol{c}\boldsymbol{3}+\boldsymbol{v} \right)+\boldsymbol{a}+\boldsymbol{s}\boldsymbol{2}=120+\boldsymbol{10}+\boldsymbol{2}=132 s$$

$$VF duration=Interval bewteen two shocks-\boldsymbol{r}=132-\boldsymbol{60}=72 s$$

Equation 15 : Detailed calculation of ventricular fibrillation duration for shockable rhythm considering the 2010-2020 guidelines

1. **Future?**
   1. ***Non-Shockable***

A possibility for adult OHCA for the coming years could be to analyse rhythm during CPR, with CPR consisting of 30 chest compressions followed by 2 ventilations and a compression rate of 120/min. Figure 13 is a representation of several cycles in case of non-shockable rhythm.

**
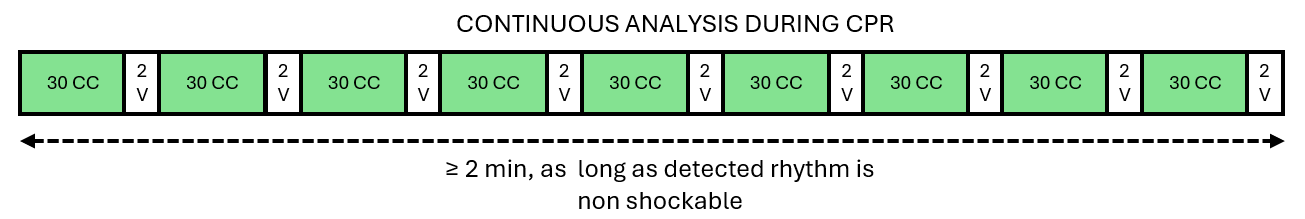
**

Figure 13 : Representation of a new proposition of analysis/CPR cycle in steady state, for non-shockable rhythms. CC: Chest Compression; V: Ventilation

**The estimated CCF for non-shockable rhythm with the new proposition of analysis/CPR cycle is 75 %** (see Equation 16 below).

$$CC duration=\boldsymbol{c}\boldsymbol{3}=\boldsymbol{15} s$$

$$Cycle duration\boldsymbol{=}\boldsymbol{c}\boldsymbol{3}+\boldsymbol{v}=\boldsymbol{15}+\boldsymbol{5}=20 s$$

$$CCF=\frac{CC duration}{Cycle duration}=\frac{15}{20}=75 \%$$

Equation 16 : Detailed calculation of chest compression fraction for non-shockable rhythm considering the new proposition of analysis/CPR cycle in steady state

- 1. ***Shockable***

A possibility for the coming years could be to analyse rhythm during CPR, with CPR consisting of 30 chest compressions followed by 2 ventilations and a compression rate of 120/min. CPR should be paused when a shockable rhythm is detected to deliver a shock to the victim and resumed immediately afterward. Figure 14 is a representation of a cycle.

**
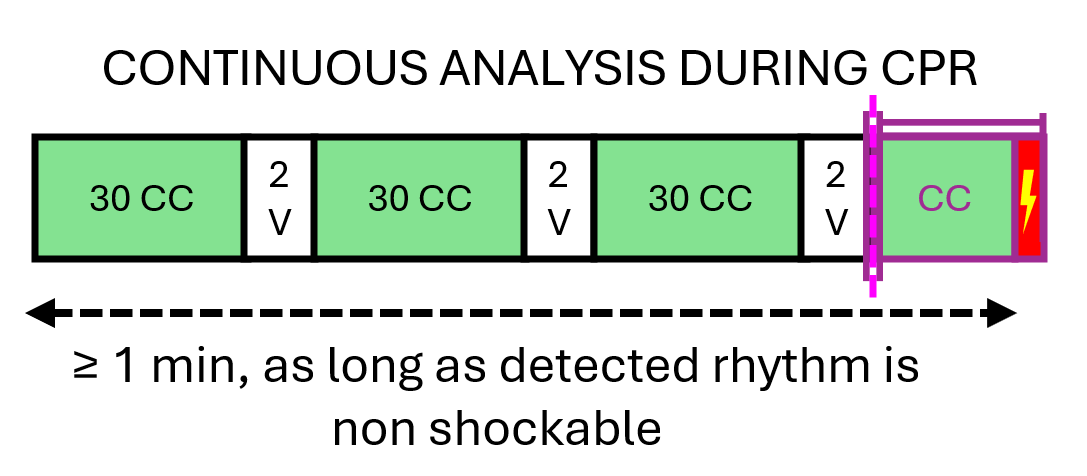
**

Figure 14 : Representation of a new proposition of analysis/CPR cycle in steady state, for shockable rhythms. CC: Chest Compression; V: Ventilation

**The estimated CCF for shockable rhythm with the new proposition of analysis/CPR cycle is between 75 and 78 %** (see Equation 17 below).

$$3*(\boldsymbol{c}\boldsymbol{3}+\boldsymbol{v}\boldsymbol{)}=60 s=\boldsymbol{r}$$

$$CPR number=3$$

$If \boldsymbol{d}= \boldsymbol{d}_{\boldsymbol{min}}$:

$${Cycle duration}_{min}=\boldsymbol{r}+\boldsymbol{d}_{\boldsymbol{min}}+\boldsymbol{s}\boldsymbol{2}=\boldsymbol{60}+\boldsymbol{5}+\boldsymbol{2}=67 s$$

$${CC duration}_{min}=CPR number*\boldsymbol{c}\boldsymbol{3}+\boldsymbol{d}_{\boldsymbol{min}}=3*\boldsymbol{15}+\boldsymbol{5}=50 s$$

$${CCF}_{min}=\frac{{CC duration}_{min}}{{Cycle duration}_{min}}=\frac{50}{67}=0.75$$

$If \boldsymbol{d}= \boldsymbol{d}_{\boldsymbol{max}}$:

$${Cycle duration}_{max}=\boldsymbol{r}+\boldsymbol{d}_{\boldsymbol{max}}+\boldsymbol{s}\boldsymbol{2}=\boldsymbol{60}+\boldsymbol{15}+\boldsymbol{2}=77 s$$

$${CC duration}_{max}=CPR number*\boldsymbol{c}\boldsymbol{3}+\boldsymbol{d}_{\boldsymbol{max}}=3*\boldsymbol{15}+\boldsymbol{15}=60 s$$

$${CCF}_{max}=\frac{{CC duration}_{max}}{{Cycle duration}_{max}}=\frac{60}{77}=0.78$$

$$CCF\in\left[ 75-78 \right] \%$$

Equation 17 : Detailed calculation of chest compression fraction for shockable rhythm considering the new proposition of analysis/CPR cycle in steady state

**The estimated VF duration for shockable rhythm with the new proposition of analysis/CPR cycle is between 7 and 17 s** (see Equation 18 below).

$If \boldsymbol{d}= \boldsymbol{d}_{\boldsymbol{min}}$:

$${Interval between two shocks}_{min}=\boldsymbol{r}+\boldsymbol{d}_{\boldsymbol{min}}+\boldsymbol{s}\boldsymbol{2}=\boldsymbol{60}+\boldsymbol{5}+\boldsymbol{2}=67 s$$

$${VF duration}_{min}={Interval between two shocks}_{min}-\boldsymbol{r}=67-\boldsymbol{60}=7 s$$

$If \boldsymbol{d}= \boldsymbol{d}_{\boldsymbol{max}}$:

$${Interval between two shocks}_{max}=\boldsymbol{r}+\boldsymbol{d}_{\boldsymbol{max}}+\boldsymbol{s}\boldsymbol{2}=\boldsymbol{60}+\boldsymbol{15}+\boldsymbol{2}=77 s$$

$${VF duration}_{max}={Interval between two shocks}_{max}-\boldsymbol{r}=77-\boldsymbol{60}=17 s$$

$$VF duration\in\left[ 7-17 \right] s$$

Equation 18 : Detailed calculation of ventricular fibrillation duration for shockable rhythm considering the new proposition of analysis/CPR cycle

**Bibliography**

[1] T. Yu *et al.*, “Adverse outcomes of interrupted precordial compression during automated defibrillation,” *Circulation*, vol. 106, no. 3, pp. 368–372, Jul. 2002, doi: 10.1161/01.cir.0000021429.22005.2e.

[2] R. Whitfield, M. Colquhoun, D. Chamberlain, R. Newcombe, C. S. Davies, and R. Boyle, “The Department of Health National Defibrillator Programme: analysis of downloads from 250 deployments of public access defibrillators,” *Resuscitation*, vol. 64, no. 3, pp. 269–277, Mar. 2005, doi: 10.1016/j.resuscitation.2005.01.003.

[3] D. Snyder and C. Morgan, “Wide variation in cardiopulmonary resuscitation interruption intervals among commercially available automated external defibrillators may affect survival despite high defibrillation efficacy,” *Crit. Care Med.*, vol. 32, no. 9 Suppl, pp. S421-424, Sep. 2004, doi: 10.1097/01.ccm.0000134265.35871.2b.

[4] S. Özlü *et al.*, “Comparison of carotid artery ultrasound and manual method for pulse check in cardiopulmonary resuscitation,” *The American Journal of Emergency Medicine*, 2023, Accessed: Oct. 02, 2023. [Online]. Available: https://www.sciencedirect.com/science/article/pii/S0735675723002954

[5] S. Savastano *et al.*, “Comparative performance assessment of commercially available automatic external defibrillators: A simulation and real-life measurement study of hands-off time,” *Resuscitation*, vol. 110, pp. 12–17, 2017.

[6] A. R. Panchal *et al.*, “Part 3: Adult Basic and Advanced Life Support: 2020 American Heart Association Guidelines for Cardiopulmonary Resuscitation and Emergency Cardiovascular Care,” *Circulation*, vol. 142, no. 16_suppl_2, pp. S366–S468, Oct. 2020, doi: 10.1161/CIR.0000000000000916.

[7] R. W. Koster *et al.*, “European Resuscitation Council Guidelines for Resuscitation 2010 Section 2. Adult basic life support and use of automated external defibrillators,” *Resuscitation*, vol. 81, no. 10, pp. 1277–1292, Oct. 2010, doi: 10.1016/j.resuscitation.2010.08.009.

[8] S. G. Beesems, L. Wijmans, J. G. P. Tijssen, and R. W. Koster, “Duration of Ventilations During Cardiopulmonary Resuscitation by Lay Rescuers and First Responders: Relationship Between Delivering Chest Compressions and Outcomes,” *Circulation*, vol. 127, no. 15, pp. 1585–1590, Apr. 2013, doi: 10.1161/CIRCULATIONAHA.112.000841.

[9] B. J. Telesz, E. P. Hess, E. Atkinson, and R. D. White, “Recurrent ventricular fibrillation: Experience with first responders prior to advanced life support interventions,” *Resuscitation*, vol. 88, pp. 138–142, Mar. 2015, doi: 10.1016/j.resuscitation.2014.10.010.

[10] V. H. T. Ha, S. Travers, D. Jost, M. Bignand, and J.-P. Tourtier, “Is time to recurrence of ventricular fibrillation a constant?,” *Resuscitation*, vol. 93, pp. e9–e10, Aug. 2015, doi: 10.1016/j.resuscitation.2015.04.034.

[11] J.-P. Didon, S. Ménétré, I. Jekova, T. Stoyanov, and V. Krasteva, “Analyze Whilst Compressing algorithm for detection of ventricular fibrillation during CPR: A comparative performance evaluation for automated external defibrillators.,” *Resuscitation*, vol. 160, pp. 94–102, Mar. 2021, doi: 10.1016/j.resuscitation.2021.01.018.

[12] F. Fumagalli, A. E. Silver, Q. Tan, N. Zaidi, and G. Ristagno, “Cardiac rhythm analysis during ongoing cardiopulmonary resuscitation using the Analysis During Compressions with Fast Reconfirmation technology,” *Heart Rhythm*, vol. 15, no. 2, pp. 248–255, Feb. 2018, doi: 10.1016/j.hrthm.2017.09.003.

[13] Y. Hu *et al.*, “The performance of a new shock advisory algorithm to reduce interruptions during CPR,” *Resuscitation*, p. S0300957219305490, Aug. 2019, doi: 10.1016/j.resuscitation.2019.07.026.

[14] C. de Graaf *et al.*, “Analyzing the heart rhythm during chest compressions: Performance and clinical value of a new AED algorithm,” *Resuscitation*, vol. 155, p. S2, Oct. 2020, doi: 10.1016/j.resuscitation.2020.08.021.
